# Design of the EBE-ST Questionnaire among Nursing Students: Multicenter Study from Eight Universities in Spain

**DOI:** 10.3390/ijerph18116145

**Published:** 2021-06-07

**Authors:** Ana Rosa Alconero-Camarero, Carmen Sarabia-Cobo, Montserrat Antonín-Martin, Alicia Borras-Santos, Montserrat Edo-Gual, Vicente Gea-Caballero, José Luis Gómez-Urquiza, José Rafael González-López, María Antonia Martínez-Momblán, Alfonso Meneses-Monroy, Montserrat Montaña-Peironcely, Diego Serrano-Gómez, Azucena Santillán-García

**Affiliations:** 1 IDIVAL Nursing Research Group, Department of Nursing, University of Cantabria, Avenida Valdecilla s/n, 39005 Santander, Spain; alconear@unican.es; 2 Escola Universitària d’Infermeria, Escoles Universitàries Gimbernat, Universitat Autònoma de Barcelona Avinguda de la Generalitat, Sant Cugat del Vallès, 08174 Barcelona, Spain; montserrat.antonin@eug.es (M.A.-M.); alicia.borras@eug.es (A.B.-S.); montserrat.edo@eug.es (M.E.-G.); 3 Nursing School La Fe, Adscript Center of University of Valencia, Research Group GREIACC, Health Research Institut La Fe, Pabellon Docente. Torre H. Avinguda de Fernando Abril Martorell, 106, 46026 Valencia, Spain; gea_vic@gva.es; 4 Facultad de Ciencias de la Salud, Universidad de Granada, Avenida de la Ilustración 60, 18071 Granada, Spain; JLGURQUIZA@ugr.es; 5 Department of Nursing, Faculty of Nursing, Physiotherapy and Podiatry, Universidad de Sevilla, C/Avenzoar 6, 41009 Seville, Spain; joserafael@us.es; 6 Escola d’Infermeria, Facultat de Medicina i Ciències de la Salut, Campus Bellvitge, Universidad de Barcelona, Carrer de Casanova, 143, 08036 Bellvitge, Spain; mmartinezmo46@ub.edu; 7 Facultad de Enfermería, Fisioterapia y Podología, Universidad Complutense de Madrid, Plaza de Ramón y Cajal, 3, 28040 Madrid, Spain; ameneses@enf.ucm.es; 8 Grup Recerca d’Infermeria, Institut d’Investigació i Innovació Parc Taulí (I3PT), Parc Taulí Hospital Universitari, Universitat Autònoma de Barcelona, Parc del Taulí, 08208 Sabadell, Spain; MMontaNa@tauli.cat; 9 Facultad de Ciencias de la Salud, Universidad de Burgos, Paseo de los Comendadores, s/n, 09001 Burgos, Spain; dserrano@ubu.es; 10 Hospital Universitario de Burgos, Av. Islas Baleares, 3, 09006 Burgos, Spain; ebevidencia@gmail.com

**Keywords:** nursing students, evidence-based nursing, evidence-based practice, nursing education research, validity and reliability

## Abstract

Background: Twenty years after the degree in nursing was introduced in Spain, the subject of evidence-based nursing is still unstructured and unestablished in most faculties. Moreover, there are hardly any rigorous studies at a national level that evaluate the current state of this competence in our faculties. Understanding the starting point is essential for the curricular design to ensure that evidence-based practice is implemented among future professionals. Aim: To design and validate an evidence-based nursing competency questionnaire for fourth-year nursing students. Methods: A specific questionnaire was developed and validated (EBE-ST). A cross-sectional survey design with psychometric validation of an instrument. Participants were 304 senior year nursing students from eight universities in Spain (2020). Results: The EBE-ST questionnaire is composed of 33 items that determine eight factors. It presents adequate reliability and validity (alpha = 0.882), measuring knowledge, attitudes and the practical application of evidence-based practice. Conclusions: We have created an instrument with good psychometric properties to measure evidence-based practice competence among senior nursing students. The heterogeneity of knowledge regarding evidence-based nursing in our country suggests that further reflection is warranted on the incorporation of this topic during undergraduate training. We have designed and validated an evidence-based nursing competency questionnaire specific to nursing students.

## 1. Introduction

Florence Nightingale (1850) was the first research nurse to develop empirical knowledge to improve nursing practice. Today, research studies continue to be key for the application of evidence-based practice (EBP) [1].

Nursing is an eminently practical profession; therefore, research is basic to develop and improve the knowledge that professionals use to perfect clinical practice. Thanks to research, empirical knowledge can be obtained to improve nursing care, patient health outcomes, and the health system [2]. Evidence-based practice is developed by integrating the best research evidence in the clinical setting together with the needs and values of patients [3]. In addition, the International Council of Nurses [4] describes EBP as a problem-solving guide for decisions made in clinical practice, involving current solid evidence, clinical experience, holistic assessment, and consideration of patient preferences. The integration of these elements facilitates safe care with positive patient outcomes, optimizes nursing time and reduces healthcare costs [3]. In addition, professional autonomy and job satisfaction are promoted and potential benefits are obtained for patients and the healthcare system [5]. For this reason, evidence-based nursing (EBN), which is embedded in EBP, has become a basic knowledge in the planning and implementation of health systems worldwide. As EBN develops, it replaces certain traditional models of health care authority in decision making. Health professionals have an obligation to keep their knowledge up to date so that they can apply it correctly during practice [1].

Nurses must not only demonstrate knowledge but also apply research findings such as the implementation of evidence-based protocols and guidelines and display a critical attitude. Without these skills, nurses will have difficulty improving their clinical practice [6]. However, researchers such as Yoo et al. [7] indicate that nurses may not be well educated to apply EBN.

Therefore, the acquisition of competence in EBN is a challenge for students who will become future nurses. In the United States, the United Kingdom, Australia and some European countries, the nursing profession has been highlighted via EBP, and active movements developed by specialized organizations such as the Cochrane and the Joanna Briggs Institute [8]. In Spain, within the framework of the Reform of the Spanish University System and the European Space for Higher Education, a degree in Nursing has been established. Its objectives include ensuring that undergraduate students acquire knowledge and skills on basic aspects of research methodology (Order CIN/2134/2008) [9]. In this sense, in some faculties, efforts are being made to incorporate a subject that integrates competency in EBN into the curricula [10]. However, there are very few publications on the introduction of competency in EBN among nursing students and in each faculty, this is carried out in a different and partial way, normally integrated within other subjects. Furthermore, two more difficulties arise, all of which can be overcome. The first is that faculty members and clinical associate professors may not have sufficient knowledge to teach this type of subject and to support students at health care centers. The second is that students may not be sufficiently involved in the learning of EBP [11].

There is a growing literature investigating this subject among undergraduate nursing students [12,13,14] particularly in relation to the tools needed for assessment purposes, which, according to Ruzafa-Martínez et al. [10] and Upton et al. [15] are clearly insufficient for not being specific for students. These authors present two questionnaires that measure similar factors, although the S-EBPQ by Upton et al. [15] has an additional benefit, as it compares knowledge and application of EBP in all levels of practice, including the student, the recent graduate, and the nurse practitioner. These are appropriate but not specific instruments for nursing students. The student is in a formative period and his or her learning of EBE is slightly different from that of a recently graduated professional, who has some basic knowledge and who is expected to apply EBE to his or her clinical practice. The lack of instruments that specifically evaluate the application of EBE in clinical practice and in theoretical classes during the course is necessary to be able to evaluate the student’s learning.

Twenty years after the degree in Nursing was introduced in Spain, the subject of EBN is still unstructured and unestablished in most faculties. Moreover, there are hardly any rigorous studies at a national level that evaluate the current state of this competence in our faculties. Understanding the starting point is essential for the curricular design to ensure that evidence-based practice is implemented among future professionals.

The principal aim was to design and validate an evidence-based nursing competency questionnaire for fourth-grade nursing students. The secondary aim was to analyze the EBN competence of students from the participating universities.

## 2. Materials and Methods

A multi-center cross-sectional design, with psychometric validation of a questionnaire in Spanish. The data were collected from January to April 2020.

### 2.1. Sample

Students in the final year of the Nursing Degree from eight universities in Spain. The inclusion criteria were voluntary participation. Sample selection was intentional. For a sample universe of 1217 students, a sample of 266 individuals was sufficient to estimate, with 95% confidence and a margin of error of 5, a representative population mean.

### 2.2. Variables

Sociodemographic variables were gathered (age, gender, previous degrees, if currently working).

A specific instrument was created to collect all the characteristics of the study of knowledge and the attitudes of nursing students towards EBN known as the EBE-Student (EBE-ST).

### 2.3. Instrument

#### 2.3.1. Design of the Questionnaire

The questionnaire was designed in two phases: in the first phase, an extensive literature review was conducted for the selection of items, in the second phase it was submitted to the criteria of a panel of three EBN experts (nursing professors specializing in EBN) for the review of content validity, after which the relevant modifications were carried out. The Delphi Method and the Kendall concordance test were used.

Brainstorming sessions were held in which the selected experts made proposals for the characteristics or attributes that should form part of the questionnaire. This step was carried out twice, giving rise to a series of suggestions and changes, leading to the final questionnaire, consisting of 40 items with a Likert-type response with five levels (totally disagree, disagree, indifferent, agree, totally agree). An initial pilot study was conducted with 20 students who provided suggestions regarding the understanding of the items, time spent, etc. The pilot study led to some major changes in the formulation of the questions, and the final version of the questionnaire, known as the EBE-Student (EBE-ST), was developed with 38 items.

#### 2.3.2. Procedure

The questionnaire was implemented on an online platform, using the online Google Forms tool to create surveys. A cover letter was prepared with the request for students’ collaboration, explaining the purpose of the study, a description of the questionnaire (including the number of items and the estimated time required for completing the questionnaire) and information concerning the project’s research team, as well as a link to access this information. The anonymity of the responses provided was guaranteed and the results were uploaded into a database for subsequent analysis. The questionnaires were sent out by convenience sampling via a mailing list prior to authorization from the participating centers. Each center was assigned a researcher who was responsible for sending all students the questionnaire based on the mailing list.

### 2.4. Ethical Considerations

The approval of the Ethics Committee of Projects of the University of Cantabria, Spain, was obtained (CE 10/2019) as well as the authorization of all the participating centers. The students gave their consent after reading the information related to the study, through the link provided by the research professor at each university. The data were anonymous, the participation was voluntary and had no impact whatsoever on the academic training of the students.

### 2.5. Statistical Analysis

Analysis of the reliability and validity of the questionnaire. The construct validity of this questionnaire was calculated using a factorial analysis by extracting principal components and Varimax rotation with Kaiser. Each variable was included in a single factor, according to its factor load, establishing values of 0.50 as a minimum saturation criterion. The Varimax rotation is assumed to be the most appropriate, since it is expected to discriminate the maximum of factors that form the scale. The Kaiser–Meyer–Olkin sample adequacy (KMO; range 0–1) and Bartlett statistical significance estimators were calculated (if the value obtained is close to one and it is significant *p* < 0.05, this indicates that the analysis with reduction of variables is adequate). The reliability of the questionnaire was calculated based on the analysis of internal consistency, for which the Cronbach’s Alpha coefficient was used, which should be interpreted as an indicator of the internal consistency of the items, since it is calculated based on the covariance between them. For the descriptive analysis of the sample, the means and standard deviations were calculated for each item, a correlation study (Pearson) was conducted between the sociodemographic variables and the items, and a descriptive study of the total of the questionnaire was made for each participating university. A multifactorial ANOVA was calculated to analyze possible differences between the universities (the university was used as the independent variable). The statistical package IBM SPSS Statistics 22 was used. A bilateral contrast and a 95% confidence level were adopted.

## 3. Results

### 3.1. Reliability and Validity of the EBE-ST Questionnaire

A total of 304 questionnaires were collected from a total sample of 1217 students for a response rate of 24%. The mean age of the students was 24.04 (SD +/− 5.95) and 81.3% (n = 244) were women. Up to 95.3% (n = 286) did not hold any other degrees and 41% (n = 123) worked while studying.

### 3.2. Analysis of the Reliability and Validity of the Questionnaire

Kaiser–Meyer–Olkin sample adequacy estimators (KMO = 0.841) and Bartlett statistical significance estimators (*p* < 0.001) were calculated. The analysis of communalities indicated that all items were above 0.5, and therefore all questions were accepted as valid for inclusion in the questionnaire. Using the principal components method, 12 principal components or factors were obtained with their own values greater than 1, which explain 65.89% of the total explained variance, a percentage of explanation that meets adequate levels of acceptance. Each item was included in a single factor, according to its factorial load, establishing values of 0.30 as a minimum saturation criterion. The factorial solutions rotated according to VARIMAX, formed a well-defined structure without overlaps. Table A1 shows the matrix of rotated components of the 38 items of the scale with their respective factor loadings for each of the 12 factors.

See Table A1. Rotated Component Matrix.

Only one item did not meet the threshold value of 0.3, and therefore it was removed (At break time we have other priorities and we don’t bring up the subject of EBN). Two factors only have one item: Factor 8 (In clinical practices, during our break we discuss queries about research and the application of care) and Factor 12 (I have been taught in theory classes that the highest levels of evidence coincide with the highest degrees of recommendation). The literature suggests that factors are considered well-defined when at least three variables have their highest weight in the same (Kim and Mueller, 1994; Costello and Osborne, 2005), therefore, there is no point discussing a factor formed by only one item: this would mean that this item is not specially related to the other factors, and therefore we decided to remove these items as well. At least two variables are needed to discuss something common. A solid factor would be defined by approximately five items with weights of 0.50 or more in the factor. For this reason, we also decided to discard the factors that had only two items (such as Factors 6 and 7, with two and three items respectively). However, with the item “I consider EBN necessary to perfect my skills”, an exception was made, and it was finally kept, as it saturated above 0.3 in Factor 2. In this manner, a questionnaire was chosen with 33 items that saturated in 8 factors, which explain 68.87% of the total variance explained.

To evaluate the internal consistency of this 33-item questionnaire, a Cronbach’s alpha value for the questionnaire of 0.882 was obtained. This value was not improved by eliminating any of the items, nor the two items that saturate in a single factor, and therefore they were all maintained. The item-total correlation values ranged from 0.50 to 0.75.

As a result of the validation process, a final questionnaire was obtained consisting of 33 items that determine eight factors that have been identified as:

Factor 1. Searching for the best evidence.
Factor 2. Ability to interpret and use.
Factor 3. EBN applied to clinical practices.
Factor 4. Knowledge of EBN during the degree.
Factor 5. EBN used by professionals.
Factor 6. EBN and profession
Factor 7. Professional future and performance.
Factor 8. EBN and clinical practice.

The questionnaire has proven to have a good factorial structure, a good predictive capacity and high internal consistency.

### 3.3. Descriptive Analysis of the Results

Table 1 shows the means and standard deviations, calculated from the Likert scores (from 1 to 5, with an increasing degree of agreement on the item) for each of the 33 items selected for inclusion in the EBE-ST questionnaire, in descending order, with the items with the greatest degree of agreement among students appearing first. No significant correlations were found between the variables age, sex, previous studies or current job and any of the factors of the questionnaire.

As displayed in Table 1, 15 items scored above 3.5 and 18 items scored below 3. The total score obtained in the questionnaire by each participating university was also analyzed (Table 2). The total score was obtained by adding the score of each item and calculating the mean, and therefore, the higher the score, the greater the competence in EBN. All obtained values were located in the first quartile (the range of the instrument ranges between a minimum of 33 and a maximum of 165, where a higher score indicates more competence in EBN). No statistically significant differences were found among the universities (*p* = 0.08).

## 4. Discussion

The principal aim of the study was to create an instrument with good psychometric properties for evaluating the competence of EBP among fourth-year nursing students from eight Spanish universities, presenting a first multi-center assessment since the establishment of the degree. The questionnaire is robust in terms of reliability and validity, with eight factors that explore the most outstanding aspects of EBN, according to the literature [16], related to attitudes, knowledge and skills in the application of EBN. The sample was homogeneous by university in terms of the total results of the questionnaire, which has allowed to have a sample, that is suited for measuring the psychometric properties of the instrument, which favors its use among senior nursing students in Spain.

As for the results concerning the students’ score obtained on the questionnaire, it is interesting to note the dichotomy found between the positive attitude referred to by students in their final year of study and their lack of knowledge, scoring less on the items specifically evaluating knowledge. This may be partly justified by the heterogeneity of the sample, since each faculty allocates an unequal number of hours to different EBN subjects and to research, which is consistent with similar studies carried out elsewhere [13,17]. The positive attitude shown by these students is noteworthy and suggests that they will apply EBP in their professional practice, which is a valuable finding in itself. However, the data from our study show that students have shortcomings in terms of their overall use of scientific literature and their ability to measure the quality of research, both of which are considered basic for the proper application of the EBP. These results coincide with a recent meta-analysis [16]. Our results suggest that the EBE-ST questionnaire is an interesting strategy to analyze the knowledge and attitudes that the student refers to possess, however, it would be interesting to compare this with the practical reality of the student’s behavior. To do so, it would be necessary to collect evidence of this knowledge and attitudes in a more applied or practical manner, such as through the resolution of a clinical case with scientific evidence or the evaluation of clinical practices through the application of competencies based on EBN on behalf of students. It is important to remember that behavior requires prior knowledge and attitudes as a modulator of learning [18].

Clinical learning environments are an ideal place to apply EBN and see students in action since it is known that learning through modeling is one of the most powerful methods of acquiring attitudes and behaviors [19,20]. Even so, teachers and nurse educators cannot assume that students arrive prepared to apply EBN knowledge and skills in clinical practice. On the other hand, it is worth mentioning that the participants in our study, at the time of answering the questionnaire, had already performed about 70% of their clinical practices, which makes us think that they were able to perceive the application of EBN in the healthcare environment. The low scores on some items, both knowledge and attitudes, can be justified, in part by the insufficient use of EBN among nurses in their regular practice, as highlighted by a recent study in the USA with 2344 nurses from 19 hospitals or healthcare systems who completed a survey [21]. Similar results are found in other countries such as China [22]. A recent systematic review suggests that to truly improve nurses’ application of EBP, objective measurements should be used as the standard when testing interventions aimed at advancing the knowledge, skills, and capacity of EBP [23].

Therefore, it seems clear that further development and involvement of clinical nurses are required to enable students to develop a clear understanding of how to carry these skills forward into their future careers [10,12].

The results of the present study can contribute to expand the nomological network and the theoretical framework of the EBN construction, although always with the caution of the limitations of a cross-sectional design. However, the EBE_ST is a specific instrument for nursing students, which is one of the strengths of this study. Other available instruments are very complete in terms of domain structure, although they only include the measurement of the use of EBN and do not measure the workplace context or clinical practice environment [24]; or they are limited to the identification of barriers and/or facilitators for the transfer of scientific research results into practice [25]. The HS-EBP instrument [26] overcomes these limitations, however, it is geared toward registered nurses, not students. Regarding the instruments directed at students, there are those that measure the factors that influence the internalization and implementation of EBN, such as the S-EBPQ questionnaire, which measures practice, attitude toward EBN, and knowledge and skills about this subject [27] or the EBP-COQ, which uses three subscales to measure the attitude toward EBN, skills, and knowledge [10,28]. The difference in the case of the EBE_ST is in the orientation of what it measures, since EBE_ST organizes the data through the phases of EBN, which can favor the orientation of the teaching to the nursing students, identifying the areas that deserve more attention or in-depth knowledge. Another striking point is that previous studies evaluated students from all years, whereas, in our study, only fourth-year students were evaluated, which is more realistic because that is when you can best evaluate the acquisition of theoretical and practical knowledge throughout the entire degree.

### Study Limitations

There are a number of limitations related to the cross-sectional design and the sample; a convenience sample was used and therefore, the results may not be generalizable to all nursing students in Spain. The response rate from the final-year nursing students was rather low. We could consider this study as a pilot study of a useful instrument for use in more focused and hypothesis-testing studies. Another limitation was there are questionnaires that have assessed EBP in nursing students, but they are not specific to students, so this was not carried out concurrent validity with those instruments. However, this may be a limitation to this study. There is also the possibility that the students who are less motivated regarding EBP may have chosen not to respond to the questionnaire. Another aspect is related to the use of a self-administered questionnaire. The knowledge of EBP has not been evaluated directly, therefore. an overestimation of the responses is possible. However, the use of this type of design is very common and allows the comparison of the results with other similar observational studies [15].

Wider samples that include students from other countries are required, as the EBP is widely used as a basis for quality of care. It would also be interesting to conduct a longitudinal study of these students when they are practicing as professionals, in order to evaluate whether their own professional practice has in any way modified the attitudes and knowledge towards EBN they had as students. Future studies should also test models to determine which variables have the greatest influence on the competence of EBN. In addition, there is a need to investigate the application of EBN, i.e., how to most effectively integrate research, the student’s own experience, and patients’ preferences, values and capacities.

## 5. Conclusions

An instrument with good psychometric properties has been created to measure the EBN competence of fourth-year nursing students from eight Spanish universities, providing a first multi-center assessment since the establishment of the nursing degree. Nursing students present a level of EBP competencies that can be improved upon, although no significant differences in knowledge was found between different universities.

A replicable tool has been designed within a national context and may be applicable for use with online students in Spain and other countries.

This is a simple tool which evaluates both attitudes and knowledge on the application of EBN by nursing students, both during their theoretical training and in their clinical practices.

Considering the fact that in Spain there is no clearly established subject or training in EBN during nursing studies, it seems interesting to carry out a deep reflection on the curricular incorporation of EBN in nursing degree education.

## Figures and Tables

**Table 1:** Descriptive data of the questionnaire items, mean and standard deviation (N = 304).

	N	Mean	SD
When I become a graduate nurse, one of my priorities will be to meet the needs and concerns of patients and families.	298	4.66	0.66
I consider EBN to be necessary to improve my competencies.	300	4.46	0.72
EBN is central to the evolution of my clinical practices.	300	4.46	0.74
EBN makes me feel more confident in my actions as a student.	299	4.44	0.76
With the consensus of teachers responsible for clinical subjects/practices, evidence-based nursing (EBN) is a tool to assist in decision making.	300	4.3	0.80
In the places where I perform clinical practices, there are guidelines and protocols that help me in my interventions.	296	4.26	0.91
I like to be well informed about the evidence in the interventions and care we provide.	297	4.23	0.91
When students detect an incorrect intervention and discuss the reason with professionals, they often respond by saying “it has always been done this way”.	300	4.03	1.07
As a student I agree with the professionals when the patient’s results do not match up with the expectations.	295	3.98	0.97
When I have any doubts related to what has been seen in clinical practices regarding interventions and care, I try to solve them by using protocols, guidelines or scientific articles.	300	3.95	1.14
Whenever I have questions related to a health problem I consult the clinical practice guidelines.	297	3.58	1.01
When I read a study I look at the implications for clinical practice.	297	3.57	1.07
I get answers to my questions by consulting scientific articles.	298	3.57	1.09
I feel capable of searching for quality articles using the methodology explained in the classroom.	300	3.54	1.12
The professional skills of nurses are mainly obtained through lengthy experience.	298	3.52	0.97
I understand the research methodology used in the studies I read.	298	3.28	0.98
During my studies, whether in class or as an intern, we often read scientific articles.	301	3.26	1.16
During the nursing degree, much is written and spoken about research and development.	301	3.24	1.1
I admit that I don’t like reading scientific studies.	297	3.12	1.21
In my practices I always observe how professionals base their actions on evidence-based nursing.	299	3.03	1.21
The nursing profession consists mainly of practical work.	300	3.03	1.22
To solve my doubts I always search for articles in databases.	300	3.02	1.08
We observe that professionals make decisions based on EBN.	298	3	1.01
I know how to interpret the statistical results of a paper.	297	2.95	1.15
I know the confounding and limiting variables of the studies I read.	295	2.94	1.21
I am able to recognize the levels of scientific evidence of studies.	297	2.93	1.04
(In previous years) During the degree I have read many scientific articles.	301	2.92	1.21
In clinical practices, professionals make it easy for me to read scientific articles.	295	2.75	1.31
During my training in the degree I have received many hours on research.	300	2.72	1.14
I have observed that many nurses do not apply evidence-based practice because they do not assign importance to the research-based care available, therefore I believe that EBN is an issue.	295	2.72	1.29
I feel able to evaluate the quality of a scientific article.	301	2.7	1.11
Since I know the EBN methodology, whenever I have questions about care, I apply the PICO methodology.	297	2.59	1.14
The scientific language of the articles is too complicated for me.	298	2.45	0.99

**Table 2:** Score descriptors on the EBE-STUDENT questionnaire for each university *.

		N	Mean	Standard Deviation
University	U of Cantabria	21	123.11	17.11
	U of Burgos	25	120.70	18.77
	U of Sevilla	83	114.67	17.09
	U of Granada	40	121.03	11.46
	U Complutense de Madrid	41	119.23	12.87
	U Barcelona	79	114.31	15.90
	UAB Gimbernat	18	117.56	18.46
	U Valencia	37	124.39	12.05

* no statistical differences were found between the means.

## Data Availability

No appliable.

## Appendix A

**Table A1:** Rotated Component Matrix (In bold type the items that saturate in each factor.)

	Factor											
	1	2	3	4	5	6	7	8	9	10	11	12
When I have doubts related to what has been seen in clinical practices with regard to interventions and care. I try to resolve them by resorting to protocols. guidelines or scientific articles.	**0.764**	−0.02	0.061	0.051	0.155	0.011	−0.10	0.108	0.086	−0.09	0.221	0.054
Whenever I have questions related to a health problem I consult the clinical practice guidelines.	**0.741**	0.003	0.073	−0.05	0.126	0.076	0.001	−0.02	0.154	−0.00	0.043	0.198
To address my queries I always search for articles in databases.	**0.723**	0.349	0.114	0.222	0.041	0.085	−0.03	0.005	−0.02	0.037	0.058	−0.09
I get answers to my questions by consulting scientific articles.	**0.647**	0.393	0.173	0.184	0.066	0.092	0.035	0.056	0.056	0.078	−0.00	−0.06
I like to be well informed about the evidence surrounding the interventions and care we perform.	**0.613**	0.011	0.435	0.059	0.089	−0.01	−0.04	0.06	0.184	−0.00	0.116	0.129
I know how to interpret the statistical results in an article.	0.123	**0.752**	0.011	0.02	0.038	0.072	0.063	0.028	0.003	−0.01	0.176	0.012
I know the confounding and limiting variables of the studies I read.	−0.01	**0.74**	−0.06	0.128	0.056	0.097	−0.00	0.052	0.143	−0.04	−0.01	0.018
I understand the research methodology used in the studies I read.	0.12	**0.732**	0.038	−0.05	0.066	0.061	0.251	0.147	−0.04	−0.05	0.03	0.074
I feel able to evaluate the quality of a scientific article.	0.1	**0.569**	0.038	0.342	0.165	0.033	−0.16	0.138	0.039	0.059	−0.03	0.249
I am able to recognize the levels of scientific evidence of the studies.	0.256	**0.544**	−0.09	0.189	0.041	0.207	−0.14	0.019	0.018	0.024	0.038	0.391
I feel capable of searching for quality articles based on the methodology explained in the classroom.	0.276	**0.524**	0.199	0.353	0.069	0.212	0.132	−0.10	0.065	−0.06	−0.14	−0.05
When I read a study I look at the implications for clinical practice.	0.333	**0.461**	0.171	−0.07	−0.10	0.044	0.099	0.078	0.177	0.043	−0.02	0.395
With the consensus of teachers responsible for clinical subjects/practices. evidence-based nursing (EBN) is a tool to assist in decision making.	0.106	0.065	**0.777**	0.145	0.178	0.024	−0.01	0.1	0.041	−0.03	0.056	−0.02
EBN makes me feel more confident in my actions as a student.	0.221	−0.00	**0.753**	0.066	−0.04	0.029	−0.08	−0.01	0.001	−0.06	0.154	0.152
Evidence-Based Nursing (EBN) is central to the evolution of my clinical practices.	0.119	−0.04	**0.669**	0.287	0.041	0.108	−0.04	0.02	0.188	−0.09	0.185	−0.02
During my degree training I have been taught many hours of research.	0.068	0.122	0.156	**0.786**	0.131	−0.02	0.084	0.053	−0.01	−0.00	−0.01	0.199
During my studies. whether in class or as during placements. we often read scientific articles.	0.346	0.272	0.245	**0.629**	0.158	0.021	−0.00	0.219	0.004	0.029	0.016	0.028
(In previous courses) During the degree training I have read many scientific articles.	0.529	0.181	0.141	**0.53**	−0.10	0.136	−0.08	0.105	0.031	−0.05	0.122	−0.08
During the nursing degree. much is written and spoken about research and development.	0.134	0.204	0.294	**0.409**	0.313	−0.28	0.024	0.01	−0.02	0.191	−0.01	0.316
We observe that professionals make decisions based on EBN.	0.117	0.05	0.113	0.007	**0.814**	0.064	0.099	−0.02	0.029	−0.11	0.081	0.254
In my nursing placements I always observe how professionals base their actions on evidence-based nursing.	0.157	0.095	0.054	0.26	**0.644**	−0.09	0.05	0.145	0.269	0.029	0.289	−0.05
In clinical practices. professionals assist me with the reading of scientific articles	0.228	0.284	0.132	0.122	**0.568**	0.212	−0.03	0.151	0.088	0.074	−0.08	−0.09
I read scientific literature in English quite fluently.	0.117	0.18	0.114	−0.00	0.01	**0.847**	−0.00	0.063	0.091	−0.07	−0.02	0.105
I am able to write scientific literature in English with fluency.	0.116	0.212	−0.03	0.028	0.069	**0.81**	−0.04	0.129	−0.01	0.079	0.021	0.047
The professional skills of nurses are mainly obtained through lengthy experience.	−0.02	0.026	−0.08	−0.03	0.116	−0.09	**0.8**	−0.03	0.072	−0.00	0	−0.01
The nursing profession consists mainly of practical work.	0.028	0.222	−0.00	0.086	−0.02	0.007	**0.722**	−0.03	−0.08	−0.01	−0.04	−0.03
I admit that I don’t like reading scientific studies.	−0.22	−0.21	−0.15	−0.02	−0.14	0.209	**0.435**	−0.07	−0.22	0.269	−0.06	0.124
In clinical practices. during our break we discuss queries about research and the application of care.	0.152	0.136	0.035	0.189	0.137	0.152	0.052	**0.798**	0.134	0.039	0.045	0.04
When I become a graduate nurse. one of my priorities will be to meet the needs and concerns of patients and families.	0.239	−0.03	0.036	0.07	−0.14	−0.02	−0.11	0.023	**0.741**	−0.26	0.134	0.18
As a student I agree with the professionals when the patient’s outcomes are different to what is expected.	0.032	0.167	0.139	−0.05	0.186	0.06	−0.08	0.069	**0.696**	0.14	0.012	0.079
In the places where I perform clinical practices there are guidelines and protocols that help me with my interventions.	0.133	0.098	0.06	0.027	0.359	0.113	0.261	−0.15	**0.574**	−0.03	−0.11	−0.12
I have observed that many nurses do not apply evidence-based practice because they do not assign importance to the research-based care available. which is why I believe that EBN is an issue.	0.024	−0.08	−0.18	0.02	−0.01	−0.03	−0.07	−0.13	−0.09	**0.682**	−0.14	−0.04
When. as students we detect an incorrect intervention and discuss the reason with professionals. they often respond by saying “it has always been done this way”.	0.076	0.078	0.089	−0.02	−0.45	0.143	0.195	−0.04	0.099	**0.587**	0.188	−0.15
The scientific language of the articles is too complicated for me.	−0.20	−0.19	−0.15	0.047	0.196	−0.09	0.31	0.142	0.003	**0.428**	0.266	0.241
Since I know the EBN methodology. whenever I have questions about care. I apply the PICO methodology.	0.267	0.241	−0.03	0.252	0.194	−0.08	−0.09	0.219	0.133	**0.362**	0.256	0.042
A course on EBN would be interesting. but it should be very practical.	0.151	0.12	0.246	0.004	0.063	0.011	−0.04	0.006	0.022	0.024	**0.771**	0.051
I consider EBN to be necessary to improve my competencies.	0.394	0.02	0.45	0.025	0.072	−0.00	−0.00	0.063	0.026	−0.10	**0.539**	0.02
I have been taught in theory classes that the highest levels of evidence coincide with the highest degrees of recommendation.	0.072	0.168	0.11	0.16	0.136	0.122	−0.01	−0.04	0.086	−0.07	0.058	**0.683**
